# Insight Into the Molecular Mechanism of Podophyllotoxin Derivatives as Anticancer Drugs

**DOI:** 10.3389/fcell.2021.709075

**Published:** 2021-08-10

**Authors:** Hua-yang Fan, Zhuo-li Zhu, Hong-chun Xian, Hao-fan Wang, Bing-jun Chen, Ya-Jie Tang, Ya-ling Tang, Xin-hua Liang

**Affiliations:** ^1^State Key Laboratory of Oral Diseases and National Clinical Research Center for Oral Diseases, West China Hospital of Stomatology (Sichuan University), Chengdu, China; ^2^State Key Laboratory of Microbial Technology, Shandong University, Qingdao, China

**Keywords:** podophyllotoxin, podophyllotoxin derivatives, anticancer, mechanism, cycle arrest

## Abstract

Podophyllotoxin (PTOX) is a biologically active compound derived from the podophyllum plant, and both it and its derivatives possess excellent antitumor activity. The PTOX derivatives etoposide (VP-16) and teniposide (VM-26) have been approved by the U.S. Food and Drug Administration (FDA) for cancer treatment, but are far from perfect. Hence, numerous PTOX derivatives have been developed to address the major limitations of PTOX, such as systemic toxicity, drug resistance, and low bioavailability. Regarding their anticancer mechanism, extensive studies have revealed that PTOX derivatives can induce cell cycle G2/M arrest and DNA/RNA breaks by targeting tubulin and topoisomerase II, respectively. However, few studies are dedicated to exploring the interactions between PTOX derivatives and downstream cancer-related signaling pathways, which is reasonably important for gaining insight into the role of PTOX. This review provides a comprehensive analysis of the role of PTOX derivatives in the biological behavior of tumors and potential molecular signaling pathways, aiming to help researchers design and develop better PTOX derivatives.

## Introduction

Cancer remains one of the most concerning issues, affecting millions of people worldwide, and chemotherapy is the mainstay of cancer treatment approaches (Wilson et al., 2019). Although a large number of anticancer drugs have been applied in clinical practice, their application is greatly limited by side effects (Kartal-Yandim et al., 2016). Therefore, studies have been conducted to develop new anticancer therapeutic agents with low systemic toxicity and selective toxicity to cancer cells.

Natural products are already being used to develop molecules with optimal pharmacological properties against various diseases, including cancer. Podophyllotoxin (PTOX) is a well-known naturally aryltetralinlignane extracted from Podophyllum peltatum and is used as a chemotherapeutic agent for a variety of cancers (Zhang et al., 2018). In clinical treatment, PTOX derivatives such as etoposide (VP-16) and teniposide (VM-26) are used as chemotherapeutic agents, such as the management of small cell lung cancer (Clark and Slevin, 1987; Paz-Ares et al., 2019). However, the limitations of these drugs are prominent, such as poor bioavailability and drug resistance, which necessitate the development of new PTOX-based anticancer drugs. Currently, many PTOX derivatives have been developed by improving the chemical structure or by conjugation with other molecules, thus allowing to achieve better biological properties.

A large number of novel PTOX derivatives have been designed, synthesized, and used in the treatment of multiple cancers. This review aims to summarize the application of PTOX derivatives in different cancers and to comprehensively analyze the mechanisms of their anticancer functions at the molecular level, which will help researchers develop more favorable PTOX derivatives (Table 1).

**TABLE 1:** Involved cancer-related signaling pathways in PTOX derivatives treatment.

PTOX derivatives	Cancer type	Potential downstream targets	Therapeutic remarks	References
4β-amidopodophyllotoxins	Breast cancer	p53, cyclin B1, Cdk1, VEGF-A, STAT-3, ERK1/2, ERK-p, AKT-1, etc.	Induced cell cycle arrest	Kamal et al., 2013
XWL-1–48	Breast cancer	PI3K/AKT/Mdm2	Induced cell cycle arrest and cell apoptosis	Wang Y. et al., 2018
PTOX	Breast cancer	P53/PLK1	Inhibited cell proliferation, migration, and invasion; Induced cell cycle arrest and cell apoptosis	Zhang et al., 2020
PTOX-norcantharidin hybrids	Breast cancer	CDK1 and cyclin B1	Induced cell cycle arrest, microtubules depolymerization, and cell apoptosis	Han et al., 2016
PTOX	Lung cancer	c-MET kinase, cyclin B1, cdc2, and p27	Induced cell apoptosis	Oh et al., 2021
Ching001	Lung cancer	ER stress signaling pathway	Induced microtubules depolymerization	Chen et al., 2013
β-apopicropodophyllin	Lung cancer	ER stress signaling pathway and caspases	Induced microtubules depolymerization and cell apoptosis	Kim et al., 2018
4β-cinnamic acid-linked PTOX	Lung cancer	PARP, caspase-3, Bax, and Bcl-2	Induced cell cycle arrest and cell apoptosis	Kamal et al., 2015
Deoxypodophyllotoxin derivatives	Lung cancer	p53/cdc2/Bax/Bcl-2 and caspase-3	Induced cell cycle arrest	Sang et al., 2013
PTOX acetate	Lung cancer	ER stress pathways; caspase−3, −8 and, −9; beclin-1, Atg3, Atg5, Atg7, and LC3	Induced cell cycle arrest, cell apoptosis, autophagy	Choi et al., 2015b; Hong et al., 2016
HY-1	Colorectal cancer	Cdc2, Chk1, Chk2, and ATR	Induced cell cycle arrest and cell apoptosis	Zhao et al., 2013
4DPG	Colorectal cancer	Chk2/Twist1/EMT	Attenuated EMT-related proteins expression	Katoch et al., 2021
XWL-1–48	Hepatocellular carcinoma	cyclinA/Cdk2 complex; Bax/Bcl2	Induced cell cycle arrest and cell apoptosis	Wang et al., 2017
DPODO	Hepatocellular carcinoma	IL-6, NF-κB, TGF-β, and α-SMA	Lowered the inflammatory and fibrogenic factors in the liver	Sharma et al., 2019
MPTOX	Bladder cancer	TUBB3 and TOPIIA	Induced cell apoptosis	Sadeghi et al., 2015
PTOX-indirubin hybrids	Leukemia	CDK1, CDK2, JNK, AKT, Beclin1, and LC3-II	Induced cell cycle arrest, cell apoptosis, and autophagy	Wang J. et al., 2018
L1EPO	K562/A02 and KBv200	mdr-1	Anti-MDR	Chen et al., 2009
YB-1EPN	KBV200 and K562/A02	mdr-1 and bcl-2	Anti-MDR	Chen et al., 2010
4β-anilino-PTOX	KB/VCR	mdr-1	Anti-MDR	Hu et al., 2010
Pyridine acid esters of PTOX	K562/ADR	ERK1/2	Anti-MDR	Zhang et al., 2016a
Aromatic heterocyclic esters of PTOX	K562/ADR	ROS/MAPK	Anti-MDR	Zhang et al., 2016b
Ptox^*Pdp*^	Hepatocellular carcinoma	PI3K/AKT/mTOR and NF-κB/Snail	Attenuated EMT-related proteins expression	Li et al., 2019b
Ptox^*Dpt*^	Hepatocellular carcinoma	p53/PI3K/AKT/mTOR/EMT	Attenuated EMT-related proteins expression	Li et al., 2019c
SU212	Breast cancer	AMPK/HIF-1α	Regulate the Warburg effect	Tailor et al., 2021

## Podophyllotoxin and Its Derivatives

Podophyllotoxin (PTOX) is found in podophyllum resin from Podophyllum plants and is a subclass of lignans, which refer to a group of plant secondary metabolites. The PTOX structure consists of a dimeric backbone, which is formed by a β–β’-linkage between two phenylpropane units, representing a typical structure of lignans. In addition, PTOX includes all the functional groups of arylnaphthalenes lignans and is composed of five rings with four chiral centers, an aryl-tetrahydrofuran type backbone, and a trans-lactone. Therefore, PTOX and its derivatives show great potential as ideal chemical agents for the exploitation of anticancer drugs.

**FIGURE 1:**
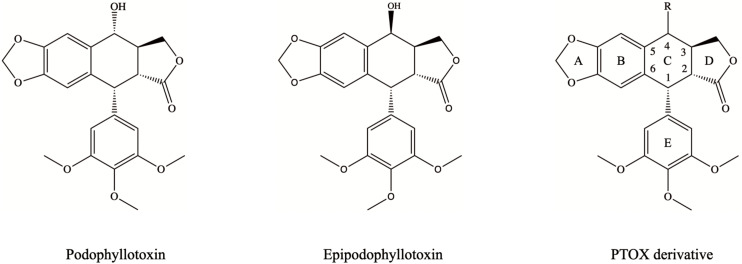
Chemical structures of podophyllotoxin, epipodophyllotoxin, and PTOX derivative.

With the development of facile synthesis methods and advances in analytical techniques such as X-ray crystallography, more and more PTOXs have been designed and developed. PTOX as a chemotherapeutic agent along with its β-configuration derivative epipodophyllotoxin are the most basic compounds used for modification to obtain better antitumor agents (Figure 1). As shown in Figure 1, the C ring and its C-4 is the major position used for conjugation with other molecule. Hybrid molecules, conjugated with other pharmacologically active molecules, may reduce the known side effects of the individual molecules and have improved anticancer ability. For instance, the poor water solubility of PTOX can be improved when hybridized with polyethylene glycol (PEG) groups (Pasut and Veronese, 2009). Plus, amino acids or peptides, which are widely used as hybrid molecules, can effectively improve the ability of drugs to cross tumor cell membranes (Hamley, 2017). Furthermore, the most common hybridization strategy is to combine PTOX with another anticancer agent, thereby obtaining better antitumor therapeutic efficacy. In this review, we highlight the significant improvement of PTOX by the hybrid molecules in the structure diagram of each compound. Such compounds can improve the specificity of antitumor drugs, reduce side effects, and override drug resistance, making hybridization an excellent strategy for the advancement of novel anticancer drugs. At the molecular mechanism level, PTOX and its derivatives exhibit significant anticancer effects by inhibiting tubulin and DNA topoisomerase II, respectively, leading to cell cycle arrest and DNA breakage (Zhao et al., 2020). In addition, PTOX derivatives have selective high toxicity against various drug-resistant tumor cells. Nowadays, mounting researchers in the study of PTOX derivatives are now focusing on the interaction between PTOX and cancer-related signaling pathways, which will help develop more selective antitumor drugs with fewer systemic side effects. A drug delivery system (DDS) loaded with PTOX derivatives was also developed to address the drawback of poor bioavailability of PTOX derivatives and to enable the controlled release of PTOX-based drugs.

## PTOX Derivatives as an Anticancer Drug for Various Cancers

The excellent antitumor activity of PTOX derivatives has been widely investigated, and plenty of novel derivatives have been exploited and their function validated in different neoplasms. In this paper, we focus on the most widely studied tumors, such as breast cancer, lung cancer, hepatocellular carcinoma, and colorectal cancer.

### Breast Cancer

**FIGURE 2:**
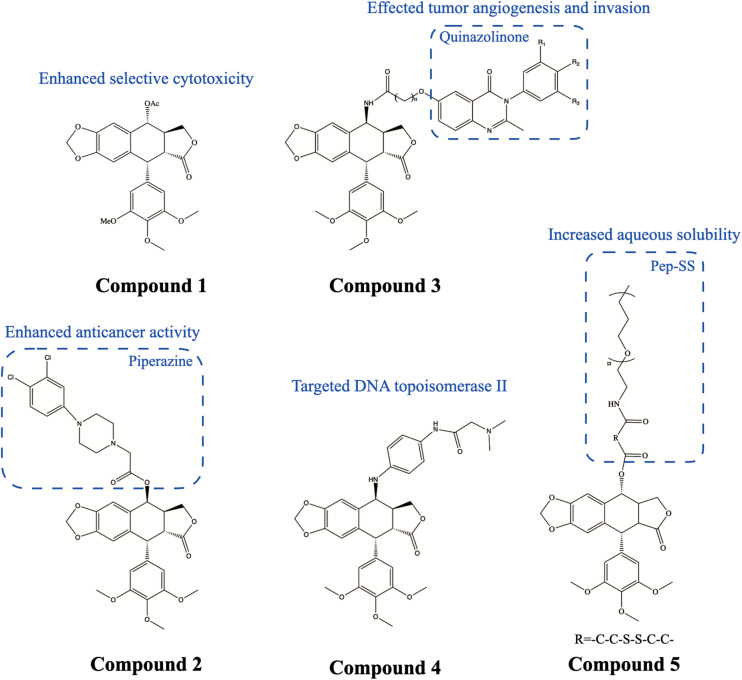
Chemical structures and therapeutic highlights of PTOX derivatives Compound 1–5.

The latest data from the International Agency for Research on Cancer (IARC) shows that breast cancer has become the most common malignancy in the world, posing a serious threat to women’s health. Triple-negative breast cancer (TNBC) is the most aggressive type of breast cancer with a high rate of metastasis and recurrence, accounting for 15–20% of all diagnosed breast cancer cases (Foulkes et al., 2010). PTOX and its derivatives can inhibit the proliferation, migration, and invasion of breast cancer *in vitro* and *in vivo*, making it a promising selective treatment (Khaled et al., 2016). Acetylpodophyllotoxin Compound 1 (Figure 2) exhibited selective inhibitory activity on human breast cancer (BT-549) cell line (Peña-Morán et al., 2016). Similarly, PTOX piperazine acetate derivatives Compound 2 (Figure 2) showed highly selective damage to the human breast cancer (MCF-7) cell line by causing G2/M blockade and microtubule disruption, while no damage was observed in non-cancerous cells (Sun et al., 2017). The 4β-amidopodophyllotoxins Compound 3 (Figure 2) induced cell cycle arrest, increased p53 and cyclin B1 protein expression, and decreased Cdk1 in MCF-7 cells, suggesting that these conjugates have an inhibitory effect on mitosis (Kamal et al., 2013). It was also observed that the expression of proteins that regulate the tumor microenvironment (e.g., VEGF-A, STAT-3, ERK1/2, ERK-p, etc.) was reduced after treatment of MCF-7 cells with these PTOX derivatives. This suggested that these PTOX derivatives may affect tumor angiogenesis and invasion. Wang Y. et al. (2018) synthesized a new orally PTOX derivative Compound 4 (Figure 2), XWL-1–48, which can bind to topoisomerase II through the formation of two strong hydrogen bonds and potential pi-pi interactions. XWL-1–48 exhibited robust antitumor activity in both *in vitro* and *in vivo* breast cancer models. It led to the production of ROS and γ-H2AX and activated the ATM/p53/p21 pathway to trigger DNA damage response in human breast cancer cells, and also induced mitochondrial apoptosis and cell cycle arrest. Zhang et al. (2020) comprehensively analyzed the molecular mechanism of PTOX on TNBC, and they suggested that PLK1 may be a downstream key gene.

Furthermore, many drug delivery system (DDS)-based products were developed to improve aqueous solubility and reduce the side effects of PTOX. Li et al. (2019a) developed redox/pH dual-sensitive nanoparticles (NPs) Compound 5 (Figure 2) by combining T7-peptide (Pep) modified polyethylene glycol (PEG) with PTOX (Pep-SS-NPs) through disulfide bonding. Compared to paclitaxel (PTX) or doxorubicin (DTX), Pep-SS-NPs showed 57—270-fold lower resistance index values against different drug-resistant cancer cell lines. *In vivo* experiments showed that Pep-SS-NPs enhanced anticancer efficacy against MCF-7/ADR xenograft tumors compared to controls.

### Lung Cancer

**FIGURE 3:**
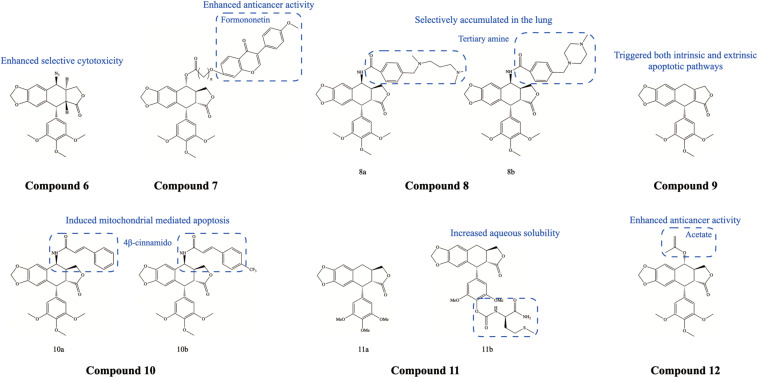
Chemical structures and therapeutic highlights of PTOX derivatives Compound 6–12.

Following breast cancer, lung cancer has the second-highest incidence rate of all cancers in the world and remains the leading cause of cancer death. Numerous studies have shown that PTOX and its derivatives can inhibit the growth of human lung cancer cells and induce apoptosis, cell cycle G2/M blockade, and ROS production (Passarella et al., 2008; Cho et al., 2016). Oh et al. (2021) investigated PTOX-induced apoptosis in human lung cancer cells and found that inhibition of c-MET kinase activity contributed to PTOX-induced cell death. Chen et al. (2013) developed a podophyllotoxin derivative Compound 6 (Figure 3), Ching001, which is specifically lethal to a variety of human lung cancer cell lines but has no significant cytotoxicity to normal human lung cell lines. The hybrids of PTOX and formononetin Compound 7 (Figure 3) displayed better inhibition of invasion and migration in human lung cancer (A549) cell line compared to the parent PTOX, and these PTOX derivatives induced apoptosis in A549 cells mainly through the caspase pathway (Yang et al., 2019). The tertiary amine-derived 4′-demethyl-epigallocatechin Compound 8 (Figure 3) adducts showed strong cytotoxicity against small cell lung cancer (SCLC) cell lines and non-small cell lung cancer (NSCLC) cell lines (Zhou et al., 2016). These PTOX-based conjugates selectively aggregated in the lung and significantly reduced toxicity to normal organs and gastrointestinal damage compared to VP-16. β-apopicropodophyllin (APP) Compound 9 (Figure 3) also showed potent anticancer activity against NSCLC cells (Kim et al., 2018). Molecularly, APP led to the accumulation of phosphorylated CHK2, p21, and phosphorylated Cdc2, disrupting microtubule polymerization and damaging DNA, and it stimulated the pro-apoptotic ER stress signaling pathway. Activation of caspase-3, -8, and -9 was also observed, suggesting that it may trigger both intrinsic and extrinsic apoptotic pathways in NSCLC cells. The 4β-cinnamic acid-linked PTOX Compound 10 (Figure 3) adduct also had a DNA topoisomerase IIα inhibitory effect on A549 cells and induced mitochondria-mediated apoptosis. At the molecular level, apoptosis was accompanied by increased expression of poly ADP-ribose polymerase (PARP), caspase-3, and Bax and decreased expression of Bcl-2 (Kamal et al., 2015). Deoxypodophyllotoxin derivatives Compound 11 (Figure 3) also inhibited microtubule formation and induced cell cycle arrest in human lung cancer cells (Sang et al., 2013). Similarly, these PTOX derivatives induced Bax expression and cleaved caspase-3, and inhibited p53/cdc2/Bax signaling and Bcl-2 expression. In addition, PTOX acetate (PA) Compound 12 (Figure 3) was found to act as a radiosensitizer in NSCLC, which may be associated with an increased rate of ROS transformation (Choi et al., 2015b; Hong et al., 2016). In addition to reducing microtubule polymerization, inducing cell cycle arrest, and causing DNA damage, PA can trigger apoptotic pathways (including caspase-3, -8, and -9 pathway) and activate pro-apoptotic ER stress pathways (increasing the expression levels of BiP, CHOP, IRE1-α, phosphorylated PERK, and phosphorylated JNK). Furthermore, PA can activate autophagy in human lung cancer cells and increase the expression of beclin-1, Atg3, Atg5, and Atg7 as well as the cleavage of LC3.

### Colorectal Cancer

**FIGURE 4:**
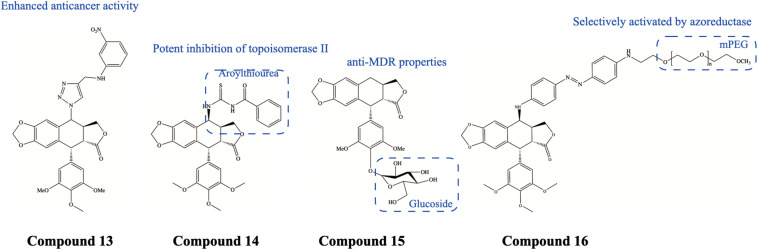
Chemical structures and therapeutic highlights of PTOX derivatives Compound 13–16.

Colorectal cancer (CRC), as one of the most common malignant diseases in developed countries, is the second leading cause of cancer death, and human CRC cell lines are commonly used in PTOX pharmacology studies. Bhat et al. (2008) synthesized 4β-[(4-substituted)-1,2,3-triazol-1-yl] PTOX derivatives Compound 13 (Figure 4) and found them to be cytotoxic against all seven human cancer cell lines, with a significant anticancer effect on human CRC (HCT-15 and 502713) cell lines in particular. Zhao et al. (2013) synthesized an aroylthiourea analog of PTOX, HY-1 Compound 14 (Figure 4) (4b-[benzoylthiourea]-4-deoxypodophyllin), which can inhibit the proliferation of human CRC (HCT116) cell lines. In addition, HY-1 can target DNA topoisomerase II and induced cell apoptosis as well as cell cycle arrest. Western blotting showed that G2/M phase arrest was associated with decreased cdc2 kinase activity and increased cdc2 phosphorylation. 5-Fluorouracil (5-FU) is a conventional chemotherapeutic agent used for the treatment of CRC, but drug resistance and recurrent cases are frequently reported (Vodenkova et al., 2020). Katoch et al. (2021) revealed that the natural PTOX derivative 4′-demethyl-deoxypodophyllotoxin glucoside Compound 15 (Figure 4) had a significant inhibitory effect on tumor growth and metastasis in CRC cells and 5-FU-resistant cells, and effectively inhibited lung metastasis in an *in situ* mouse model of CRC. Moreover, Ha et al. (2021) designed and synthesized a supramolecular hydrogel, prodrug4a Compound 16 (Figure 4), consisting of PTOX and polyethylene glycol (PEG) chains. In particular, this compound can target the colon by selectively activating azoreductase. Furthermore, this compound can act as a nanodrug carrier to deliver 5-Fu, exhibiting significant synergistic cytotoxicity against CRC cells. However, the experiment needs to be evaluated in an animal model.

### Hepatocellular Carcinoma

**FIGURE 5:**
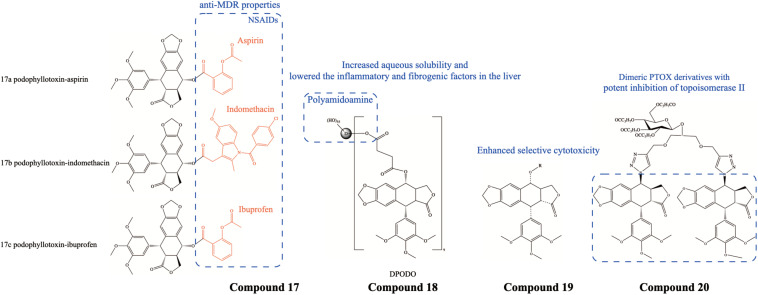
Chemical structures and therapeutic highlights of PTOX derivatives Compound 17–20.

Hepatocellular carcinoma (HCC) is the major malignancy originating from the liver, accounting for approximately 80% of all types of liver cancer (Lin et al., 2017). PTOX and its derivatives have also shown excellent therapeutic effects on HCC. Zhang et al. (2017a) synthesized PTOX conjugates with non-steroidal anti-inflammatory drugs (NSAIDs) Compound 17 (Figure 5), which showed selective toxicity against 5-FU-resistant cells. PTOX-NSAIDs disrupted their microtubule network and effectively triggered G2/M blockade as well as apoptosis. Additionally, XWL-1–48 also exerted potent antitumor activity *in vivo* and *in vitro* in HCC models (Wang et al., 2017), where it significantly inhibited the levels of the cyclinA/Cdk2 complex and increased the Bax/Bcl2 ratio at the molecular level, triggering apoptosis. Moreover, Sharma et al. (2019) synthesized a poly-amidoamine dendrimer-conjugated PTOX (DPODO) Compound 18 (Figure 5) with low water solubility and systemic toxicity, which inhibited the progression of HCC by modulating inflammatory and fibrotic factors. Specifically, at the molecular level, DPODO significantly reduced levels of IL-6 and NF-κB and decreased the expression of fibrosis markers TGF-β and α-SMA in the liver. And less fibrous tissue deposition was observed at the tissue level.

So far, most of the published studies evaluated the cytotoxic effects of PTOX derivatives on a variety of tumor cell lines. Wu et al. (2018) designed a PTOX derivative Compound 19 (Figure 5) and found it to be more cytotoxic than VP-16 and doxorubicin against lung cancer, breast cancer, and liver cancer cells. Zi et al. (2018) synthesized a novel dimeric PTOX derivative Compound 20 (Figure 5) and found it to have potent anticancer activity against leukemia, hepatoma, lung cancer, breast cancer, and colon cancer cells. In a nutshell, the excellent selective therapeutic effect of PTOX and its derivatives on a variety of tumors has attracted increasing attention. Besides the above mentioned tumors, researchers have further developed more PTOX derivatives against other different cancers as well, including pancreatic cancer (Pan et al., 2020), leukemia (Zhang et al., 2016b), head and neck squamous cell carcinoma (HNSCC) (Resendez et al., 2019), cervical carcinoma (Wang et al., 2013), etc.

## Mechanism of PTOX Derivatives as an Anticancer Drug

**FIGURE 6:**
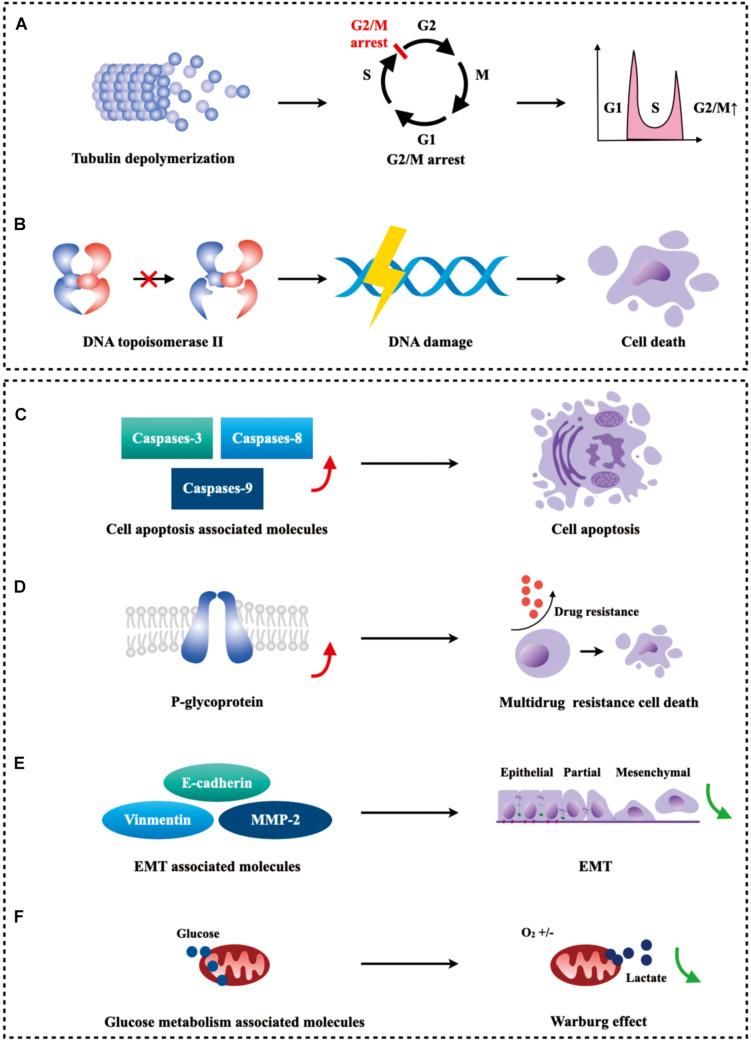
Schematic diagram of the mechanism of PTOX derivatives for cancer treatment. **(A)** PTOX derivatives act as inhibitors of tubulin to induce G2/M arrest in tumor cells. **(B)** PTOX derivatives can inhibit the degradation of topoisomerase II to cause DNA damage and eventually trigger cell death. **(C–F)** PTOX derivatives can inhibit the growth of tumor cells by affecting drug-resistant tumor cells, apoptosis-related molecules, EMT-related molecules, and gluconeogenesis-related molecules, respectively.

The recognized anticancer mechanisms of PTOX and its derivatives are as inhibitors of tubulin and DNA topoisomerase II, respectively. In addition, mounting studies on PTOX derivatives have shown that multiple cancer-associated signaling pathways may intimately correlate with their anticancer ability (Figure 6).

### Main Mechanism

#### Inhibitor of Tubulin

**FIGURE 7:**
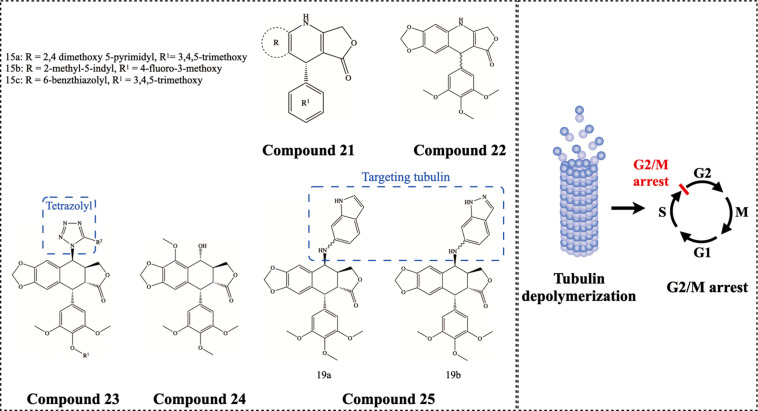
Chemical structures and therapeutic highlights of PTOX derivatives Compound 21–25.

PTOX, whose pharmacological effects have been recognized to inhibit microtubule assembly by blocking the colchicine binding site, thereby exhibiting inhibition of microtubule protein polymerization and inducing G2/M blockade, has shown potent antitumor activity (Cortese et al., 1977). PTOX derivatives have also been shown to have the analogous ability (Han et al., 2018). Kamal et al. (2011b) synthesized a series of conjugates of 4-aza-2,3-didehydropodophyllotoxins Compound 21 (Figure 7), and fluorescent tubulin polymerization analysis showed these PTOX derivatives significantly reduced tubulin units. On the molecular mechanism, these PTOX-based conjugates caused apoptosis of A549 cancer cells in a caspase-3 dependent manner (Kamal et al., 2014). Likewise, Kandil et al. (2016) synthesized 4-azapodophyllotoxin derivatives Compound 22 (Figure 7), which induces disruption of the microtubule cytoskeleton at a sub-micromolar level to MCF-7 cancer cells. Similarly, 4β-[(5-substituted)-1,2,3,4-tetrazolyl] PTOX derivatives Compound 23 (Figure 7) were synthesized, which inhibited nearly 90% polymerization of tubulin at only 5 μM concentration (Hyder et al., 2015). Sadeghi et al. (2015) investigated the antitumor effect of 6-methoxyPTOX (MPTOX) Compound 24 (Figure 7), which has a similar structure to PTOX. Treatment of human bladder cancer (5637) cell line and myeloid leukemia (K562) cell line with MPTOX significantly reduced the viability of tumor cells and induced their programmed cell death. In addition, the expression of TUBB3 (a key member of β-tubulin) and TOPIIA (a key nuclease in DNA replication) was inhibited in tumor cells after MPTOX treatment. Furthermore, Bai et al. (2012) and Zhao et al. (2015, 2019) demonstrated that the carbon-sulfur bond at the 4-position (C-4) of the carbon ring of PTOX reduced the inhibitory effect of dose on tubulin polymerization, thereby enhancing its therapeutic effect. They synthesized 4β-NH-(6-aminoindole)-4-deoxy-PTOX Compound 25 (Figure 7) that can target the α-tubulin binding site and colchicine binding domain, as confirmed by X-ray crystallographic analysis. Importantly, these PTOX derivatives exhibited nanomolar antitumor efficacy *in vitro* and no significant toxicity *in vivo*.

#### Inhibitor of DNA Topoisomerase II

**FIGURE 8:**
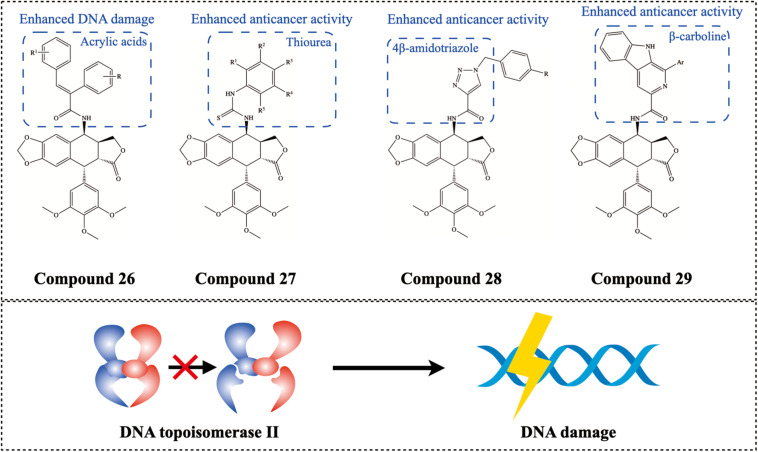
Chemical structures and therapeutic highlights of PTOX derivatives Compound 26–29.

The severe side effects of PTOX, such as high toxicity and gastrointestinal upset, have limited its use in cancer chemotherapy. Extensive structural modifications of PTOX have yielded more effective and less toxic anticancer agents, such as etoposide and teniposide, which have been widely used in cancer chemotherapy (Evans et al., 1985; Paz-Ares et al., 2019). They stabilize the cleavage complex formed between the enzyme and its DNA substrate, thereby inhibiting topoisomerase II degradation and ultimately triggering cell death through the accumulation of chromosome breaks. Topoisomerases (topo-I and topo-II) play an important role in DNA replication, transcription and are a group of promising antitumor targets. Scholars generally believe that the bulky motif at the C-4 position of PTOX may be responsible for the inhibition of topo II (Stähelin and von Wartburg, 1991), and γ-H2AX is a classical marker of double-stranded DNA breaks during DNA damage (Kinner et al., 2008). Kamal et al. (2011a) developed the new PTOX analog 4β-acrylamidopodophyllotoxin congeners Compound 26 (Figure 8) as a potential anticancer agent. Their results showed that a large number of γ-H2AX foci were observed in etoposide and this PTOX derivative-treated cells, while double-stranded DNA breaks were negligible in PTOX-treated cells. Shankaraiah et al. (2015) developed a facile one-pot method and successfully synthesized novel PTOX-thiourea hybrids Compound 27 (Figure 8), which showed selective DNA topoisomerase II inhibitory activity and anticancer activity against human prostate cancer (DU-145) cell line. In addition, the 4bβ-aminotriazole PTOX derivative Compound 28 (Figure 8) synthesized by Reddy et al. (2018) showed potent cytotoxicity on prostate cancer cell lines and was efficient in inhibiting DNA topoisomerase II. Likewise, β-carboline PTOX congeners Compound 29 (Figure 8) exhibited excellent cytotoxicity against human prostate cancer (DU-145) cell lines, and the DNA topoisomerase II inhibitory ability of these congeners was confirmed by comet analysis, DNA binding studies, and docking studies (Sathish et al., 2018).

### Potential Cancer-Associated Signaling Pathway

#### Multidrug Resistance-Associated Molecules

**FIGURE 9:**
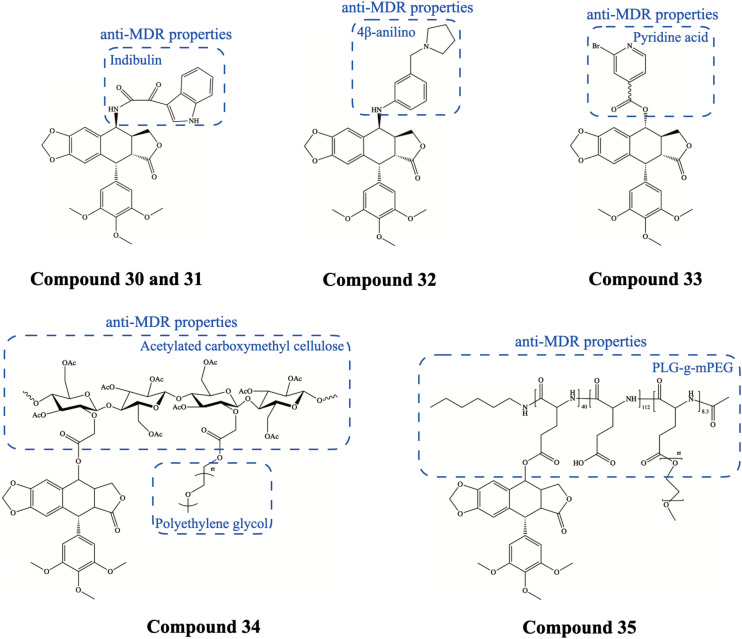
Chemical structures and therapeutic highlights of PTOX derivatives Compound 30–35.

In malignant chemotherapy, resistance to anticancer drugs can often be demonstrated and is usually manifested as multidrug resistance (MDR), which is one of the research hotspots in cancer prevention and treatment (Erin et al., 2020). The superfamily of transmembrane ATP-binding cassette (ABC) transporter proteins enhances the efflux of a wide range of chemotherapeutic agents and is regarded as one of the major causes of MDR in cancer, with the role of the highly expressed P-glycoprotein (P-gp) being the most well established (Zhang et al., 2021). Chen et al. (2009) synthesized a PTOX derivative, L1EPO Compound 30 (Figure 9), which exhibited cytotoxicity to P-gp-mediated MDR cancer cell lines (K562/A02 and KBv200) but was less cytotoxic to normal human cell lines. Furthermore, L1EPO downregulated the mdr-1 gene and reduced the expression of P-gp, which may be the mechanism of its anti-MDR effect. Similarly, YB-1EPN Compound 31 (Figure 9) as a PTOX derivative also showed cytotoxicity against KBV200 and K562/A02 cells, while downregulating mdr-1 and bcl-2 expression, suggesting its ability to overcome P-gp-mediated MDR (Chen et al., 2010). Hu et al. (2010) synthesized a range of 4β-anilino-PTOX analogs Compound 32 (Figure 9) that led to G2/M phase arrest and eventually induced apoptosis in drug-resistant KB/VCR cancer cells. In both drug-sensitive xenograft models and drug-resistant xenograft models, the administration of 4β-anilino-PTOX analogs can effectively treat cancer at lower doses than VP-16. In many other studies, it has been shown that 4β-anilino-PTOX analogs may kill MDR tumor cells (e.g., K562/A02) by downregulating mdr-1 expression (Cheng et al., 2014; Cao et al., 2015, 2018). Zhang et al. (2016a) synthesized pyridine acid esters of PTOX Compound 33 (Figure 9), which efficiently triggered cell cycle arrest and accompanied induction of apoptosis in K562/ADR tumor cells [resistant to adriamycin (ADR)]. Regarding the molecular mechanism, the results suggested that the pyridylate of PTOX may reduce the expression of the P-gp protein by stimulating the ERK1/2 signaling patway. In addition, aromatic heterocyclic esters of PTOX also showed potent anti-MDR activity against K562/ADR cancer cells, causing cellular S-phase block, apoptosis, and downregulation of P-gp expression, with molecular mechanisms suggesting an association with the ROS/MAPK pathway (Zhang et al., 2016b).

In addition, nanomedicine, which has been widely used in anti-cancer, can be used to modify PTOX derivatives to improve their poor solubility and high toxicity. Roy et al. (2015, 2017) developed PTOX-based nanomedicines by covalently coupling PTOX and polyethylene glycol (PEG) with acetylated carboxymethylcellulose (CMC-Ac) Compound 34 (Figure 9) via one-pot esterification chemistry. They prepared PTOX-based nanoparticles (NPs) with an average diameter of 20 nm, which released PTOX at ∼5%/day in serum. The NPs exhibited excellent killing ability and high selectivity for tumor cells, their accumulation in tumor tissues was eightfold higher than all other tissues, and they showed significantly improved efficacy against MDR tumors in mice with minimal systemic toxicity. Zhou et al. (2018) developed a peptide-based PTOX conjugated poly(*_*L*_*-glutamic acid)-g-methoxy poly(ethylene glycol) (PLG-g-mPEG) Compound 35 (Figure 9), which effectively inhibited the expression of P-gp in the MCF-7/ADR cancer cells. *In vivo* experiments showed that the PLG-g-mPEG-PTOX conjugate had excellent antitumor efficacy against MCF-7/ADR xenograft tumors with a tumor inhibition rate of 82.5%. Compared with free PTOX, it showed significantly higher anticancer efficacy with minimal toxicity. In addition, Feng et al. (2020) developed a pH/redox cascade-sensitive multiscale nanoparticle, PAMAM-ss-PPT, which can fully release PTOX in the presence of elevated glutathione (GSH). Results demonstrated that nanoparticles can effectively penetrate paclitaxel-resistant A549 cancer cell xenografts and inhibit tumor growth with negligible toxicity (Feng et al., 2020).

#### Cell Apoptosis-Associated Molecules

**FIGURE 10:**
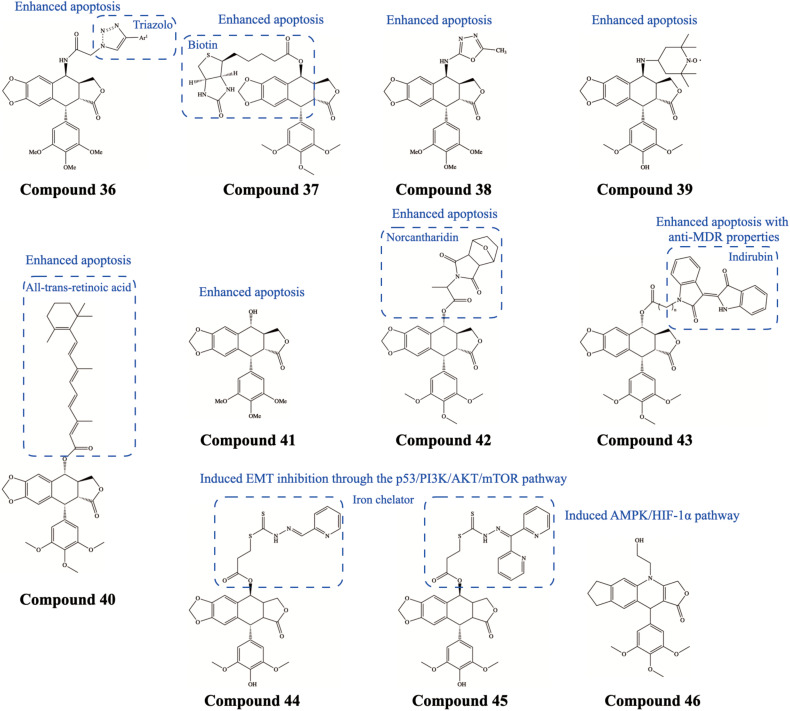
Chemical structures and therapeutic highlights of PTOX derivatives Compound 36–46.

In the studies on the antitumor function of PTOX derivatives, it was found that they can cause apoptosis and affect numerous related signaling pathways. Caspase-mediated activation is the canonical pathway for the induction of apoptosis. Precursor caspases will be proteolyzed upon receiving the apoptotic signal, and caspases-3, -8, and -9 are the most common apoptotic caspases among them (Porter and Jänicke, 1999; Kim et al., 2015; Tummers and Green, 2017). We thoroughly summarize the effects of PTOX derivatives on different cystatin caspases in cancer cells in the following (Table 2). (i) caspase-3: DPMA (Sang et al., 2013), 4-aza-2,3-didehydropodophyllotoxins (Kamal et al., 2011b, 2014), triazolo linked PTOX conjugates Compound 36 (Figure 10; Vishnuvardhan et al., 2017), deoxypodophyllotoxin (DPT) (Hui et al., 2016), biotinylated PTOX derivatives Compound 37 (Figure 10; Zi et al., 2019); (ii) caspases-8: hybrids of PTOX and formononetin (Yang et al., 2019); (iii) caspases-9: 4β-amidopodophyllotoxins (Kamal et al., 2013); (iv) caspases−3 and −9: OAMDP Compound 38 (Figure 10; Ren et al., 2018) and spin-labeled PTOX derivatives Compound 39 (Figure 10; Yang et al., 2017); (v) caspases−3, −8, and −9: β-apopicropodophyllin (Kim et al., 2018), PTOX acetate (Choi et al., 2015a,b; Hong et al., 2016), aromatic heterocyclic esters of PTOX (Zhang et al., 2016b), acid-PTOX conjugate Compound 40 (Figure 10; Zhang et al., 2017b); (vi) **multiple** caspases: picropodophyllotoxin Compound 41 (Figure 10; Kwak et al., 2020). In addition, PTOX may affect other signaling pathways to trigger cell apoptosis. Han et al. (2016) synthesized PTOX-norcantharidin hybrids Compound 42 (Figure 10), which showed low cytotoxicity against normal human embryonic kidney cells (293T) and induced cell cycle G2/M arrest and apoptosis in MCF-7 cancer cells. In terms of molecular mechanisms, the results showed an upregulation of the expression of the cell cycle-related protein CDK1 and downregulation of the expression of cyclin B1, a protein required for mitotic initiation. Wang J. et al. (2018) synthesized PTOX-indirubin hybrids Compound 43 (Figure 10), which resulted in apoptosis and cell cycle arrest in human leukemia cells (K562/VCR). Meanwhile, PTOX-indirubin hybrids elicited the accumulation of intracellular ROS, regulated JNK and AKT signaling, and downregulated the expression levels of P-gp and MRP1 proteins. On the other hand, it was revealed by Western blot that the hybrid could induce autophagy in K562/VCR cells by increasing the levels of Beclin1 and LC3-II.

**TABLE 2:** Involved caspases in PTOX derivatives treatment.

Caspases	PTOX derivatives	References
Caspase-3	DPMA, 4-aza-2,3-didehydropodophyllotoxins, triazolo linked PTOX conjugates, deoxypodophyllotoxin (DPT), and biotinylated PTOX derivatives	Kamal et al., 2011b, 2014; Sang et al., 2013; Hui et al., 2016; Vishnuvardhan et al., 2017; Zi et al., 2019
Caspas-8	Hybrids of PTOX and formononetin	Yang et al., 2019
Caspase-9	4β-amidopodophyllotoxins	Kamal et al., 2013
Caspase-3 and -9	OAMDP and spin-labeled PTOX derivatives	Yang et al., 2017; Ren et al., 2018
Caspase-3, -8, and -9	β-apopicropodophyllin, PTOX acetate, aromatic heterocyclic esters of PTOX, and acid-PTOX conjugate	Choi et al., 2015a,b; Hong et al., 2016; Zhang et al., 2016b, 2017b; Kim et al., 2018

#### Epithelial-Mesenchymal Transition Associated Molecules

Epithelial-mesenchymal transition (EMT) is a cellular program that is known to be essential for the development of malignant tumors. EMT can confer greater initiation and metastatic potential to cancer cells, as well as greater resistance to therapeutic modalities such as chemotherapy (Dongre and Weinberg, 2019). The natural PTOX analog 4′-demethyl-deoxypodophyllotoxin glucoside (4DPG), which increased the expression of checkpoint kinase 2 (Chk2), a tumor suppressor of the DNA damage checkpoint pathway. Overexpression of Chk2 eliminated the metastatic ability of tumor cells and reduced the expression of proteins associated with EMT (including Twist1, Snail1, and MMP-2) (Nayak et al., 2017). Likewise, 4DPG synthesized by Katoch et al. (2021) also enhanced the level of Chk2 and attenuated the expression of EMT-related proteins in human colorectal cancer (HCT-116 and SW-620) cell lines. Li et al. (2019b; 2019c) synthesized 2-pyridinealdehyde hydrazone dithiocarbamate S-propionate PTOX ester (Ptox^*Pdp*^) Compound 44 (Figure 10) and di-2-pyridineketone hydrazone dithiocarbamate S-propionate podophyllotoxin ester (Ptox^*Dpt*^) Compound 45 (Figure 10), both of which exhibited inhibitory effects on the proliferation, migration, and aggressiveness of hepatocellular carcinoma cells *in vitro* and *in vivo* experiments. Furthermore, in terms of molecular mechanisms, Ptox^*Pdp*^ and Ptox^*Dpt*^ may inhibit the EMT ability of hepatocellular carcinoma cells through down-regulation of the PI3K/AKT/mTOR pathway, causing a reduction of vimentin expression and an increase of E-cadherin expression.

#### Glucose Metabolism-Associated Molecules

Glucose metabolism is a key feature of tumor cells, and their proliferation is accompanied by increased demand for energy and metabolic substrates. Many cancers are adapting to these demands through a shift in metabolism termed the Warburg effect, which inhibits oxidative phosphorylation and thus promotes a glucose-to-lactate aerobic fermentation (Vander Heiden et al., 2009). A better insight into cellular metabolism may ultimately lead to superior treatments for human cancers. The effects of PTOX and its derivatives on glucose metabolism have been poorly studied. Tailor et al. (2021) synthesized a new PTOX derivative, SU212 Compound 46 (Figure 10), which exhibited selective anticancer toxicity through direct activation of AMP-activated protein kinase (AMPK). Moreover, this PTOX derivative could regulate the Warburg effect in triple-negative breast cancer cells through the AMPK/hypoxia-inducible factor 1α (HIF-1α) pathway, suggesting the potential research interest of PTOX derivatives in the field of tumor glycolysis.

## Conclusion and Perspectives

In conclusion, PTOX derivatives have shown significant anticancer properties through modification with various natural substances or nanomaterials, and some of them are already used in clinical practice. Currently, most of the molecular structure modifications of PTOX are focused on its C-4 position, and the primary function of these designed PTOX derivatives is to act as inhibitors of tubulin polymerization or topoisomerase II, thereby inducing cell cycle arrest and DNA breakage. In addition, many promising PTOX hybrids may serve as novel anticancer candidates with dual or multiple anticancer mechanisms, such as affecting MDR, apoptosis, autophagy, EMT-related pathway, and glycolysis of cancers.

However, the specific anticancer mechanism remains to be refined, which is important for the development of next-generation novel PTOX derivatives. Identification of cancer-related signaling pathways can significantly improve patient outcomes through the development of targeted novel chemotherapeutic agents. Furthermore, with the advancement of nanomedicine, novel PTOX-based drug delivery systems can be developed, and multiple therapeutic approaches can be combined. Drug delivery system approaches have been shown to significantly improve the pharmacological properties of established drugs, and several delivery technologies for chemotherapeutic drugs have entered the clinic (Manzari et al., 2021). For example, in 1995, the FDA approved the first nanoparticle drug formulation, doxorubicin-liposome, which greatly reduced the heart toxicity of doxorubicin (Gabizon et al., 2003). Also, patisiran-lipid nanoparticles, a novel nanocomplex for the treatment of amyloidosis (Clinical trial number NCT01960348), was the first siRNA therapy approved by the FDA in 2018. However, there are still very few nanomedicines entering clinical applications compared to existing studies, possibly due in part to low drug loading and delivery efficiency. With recent advances in computer-based artificial intelligence algorithms that could facilitate the development of nanoparticles, these issues may be addressed in the near future. As a natural product with multiple biological activities, the outstanding potentiality of PTOX will hopefully contribute to the development of optimal anticancer drugs.

## Author Contributions

YL-T, YJ-T, and XH-L contributed to conception and design of the study. HY-F organized the database. HY-F and HC-X wrote the first draft of the manuscript. HF-W and BJ-C wrote sections of the manuscript. ZL-Z made major contributions to manuscript revision. All authors contributed to manuscript revision, read, and approved the submitted version.

## Conflict of Interest

The authors declare that the research was conducted in the absence of any commercial or financial relationships that could be construed as a potential conflict of interest.

## Publisher’s Note

All claims expressed in this article are solely those of the authors and do not necessarily represent those of their affiliated organizations, or those of the publisher, the editors and the reviewers. Any product that may be evaluated in this article, or claim that may be made by its manufacturer, is not guaranteed or endorsed by the publisher.

## References

[B1] Bai J. K. Zhao W. Li H. M. Tang Y. J. (2012). Novel biotransformation process of podophyllotoxin to 4 β-sulfur-substituted podophyllum derivates with anti-tumor activity by *Penicillium purpurogenum* Y.J. *Tang. Curr. Med. Chem.* 19 927–936. 10.2174/092986712799034914 22214458

[B2] Bhat B. A. Reddy P. B. Agrawal S. K. Saxena A. K. Kumar H. M. Qazi G. N. (2008). Studies on novel 4beta-[(4-substituted)-1,2,3-triazol-1-yl] podophyllotoxins as potential anticancer agents. *Eur. J. Med. Chem.* 43 2067–2072. 10.1016/j.ejmech.2007.09.015 17988764

[B3] Cao B. Chen H. Gao Y. Niu C. Zhang Y. Li L. (2015). CIP-36, a novel topoisomerase II-targeting agent, induces the apoptosis of multidrug-resistant cancer cells in vitro. *Int. J. Mol. Med.* 35 771–776. 10.3892/ijmm.2015.2068 25592869

[B4] Cao B. Yang S. Li W. Chen H. Chen Y. Liu Y. (2018). GMZ-1 is a podophyllotoxin derivative that suppresses growth and induces apoptosis in adriamycin-resistant K562/A02 cells through modulation of MDR1 expression. *Mol. Med. Rep.* 17 474–478. 10.3892/mmr.2017.7862 29115592

[B5] Chen H. Bi W. Cao B. Yang Z. Chen S. Shang H. (2010). A novel podophyllotoxin derivative (YB-1EPN) induces apoptosis and down-regulates express of P-glycoprotein in multidrug resistance cell line KBV200. *Eur. J. Pharmacol.* 627 69–74. 10.1016/j.ejphar.2009.10.056 19879873

[B6] Chen H. Wang J. Zhang J. Wang Y. Cao B. Bai S. (2009). L1EPO, a novel podophyllotoxin derivative overcomes P-glycoprotein-mediated multidrug resistance in K562/A02 cell line. *Biol. Pharm. Bull.* 32 609–613. 10.1248/bpb.32.609 19336892

[B7] Chen J. Y. Tang Y. A. Li W. S. Chiou Y. C. Shieh J. M. Wang Y. C. (2013). A synthetic podophyllotoxin derivative exerts anti-cancer effects by inducing mitotic arrest and pro-apoptotic ER stress in lung cancer preclinical models. *PLoS One* 8:e62082. 10.1371/journal.pone.0062082 23646116 PMC3639983

[B8] Cheng W. H. Cao B. Shang H. Niu C. Zhang L. M. Zhang Z. H. (2014). Synthesis and evaluation of novel podophyllotoxin derivatives as potential antitumor agents. *Eur. J. Med. Chem.* 85 498–507. 25113878 10.1016/j.ejmech.2014.08.006

[B9] Cho J. H. Hong W. G. Jung Y. J. Lee J. Lee E. Hwang S. G. (2016). Γ-Ionizing radiation-induced activation of the EGFR-p38/ERK-STAT3/CREB-1-EMT pathway promotes the migration/invasion of non-small cell lung cancer cells and is inhibited by podophyllotoxin acetate. *Tumour Biol.* 37 7315–7325. 26671552 10.1007/s13277-015-4548-y

[B10] Choi J. Y. Cho H. J. Hwang S. G. Kim W. J. Kim J. I. Um H. D. (2015a). Podophyllotoxin acetate enhances γ-ionizing radiation-induced apoptotic cell death by stimulating the ROS/p38/caspase pathway. *Biomed. Pharmacother.* 70 111–118. 10.1016/j.biopha.2014.12.038 25776488

[B11] Choi J. Y. Hong W. G. Cho J. H. Kim E. M. Kim J. Jung C. H. (2015b). Podophyllotoxin acetate triggers anticancer effects against non-small cell lung cancer cells by promoting cell death via cell cycle arrest, ER stress and autophagy. *Int. J. Oncol.* 47 1257–1265. 10.3892/ijo.2015.3123 26314270 PMC4583522

[B12] Clark P. I. Slevin M. L. (1987). The clinical pharmacology of etoposide and teniposide. *Clin. Pharmacokinet.* 12 223–252. 10.2165/00003088-198712040-00001 3297462

[B13] Cortese F. Bhattacharyya B. Wolff J. (1977). Podophyllotoxin as a probe for the colchicine binding site of tubulin. *J. Biol. Chem.* 252 1134–1140. 14143

[B14] Dongre A. Weinberg R. A. (2019). New insights into the mechanisms of epithelial-mesenchymal transition and implications for cancer. *Nat. Rev. Mol. Cell Biol.* 20 69–84. 10.1038/s41580-018-0080-4 30459476

[B15] Erin N. Grahovac J. Brozovic A. Efferth T. (2020). Tumor microenvironment and epithelial mesenchymal transition as targets to overcome tumor multidrug resistance. *Drug Resist. Updat.* 53:100715. 10.1016/j.drup.2020.100715 32679188

[B16] Evans W. K. Osoba D. Feld R. Shepherd F. A. Bazos M. J. DeBoer G. (1985). Etoposide (VP-16) and cisplatin: an effective treatment for relapse in small-cell lung cancer. *J. Clin. Oncol.* 3 65–71. 10.1200/jco.1985.3.1.65 2981293

[B17] Feng W. Zong M. Wan L. Yu X. Yu W. (2020). pH/redox sequentially responsive nanoparticles with size shrinkage properties achieve deep tumor penetration and reversal of multidrug resistance. *Biomater. Sci.* 8 4767–4778. 10.1039/d0bm00695e 32724941

[B18] Foulkes W. D. Smith I. E. Reis-Filho J. S. (2010). Triple-negative breast cancer. *N. Engl. J. Med.* 363 1938–1948. 10.1056/NEJMra1001389 21067385

[B19] Gabizon A. Shmeeda H. Barenholz Y. (2003). Gabizon A, Shmeeda H, Barenholz YPharmacokinetics of pegylated liposomal Doxorubicin: review of animal and human studies. Clin pharmacokinet 42: 419-436. *Clin. Pharmacokinet.* 42 419–436. 12739982 10.2165/00003088-200342050-00002

[B20] Ha W. Zhao X. B. Zhao W. H. Tang J. J. Shi Y. P. (2021). A colon-targeted podophyllotoxin nanoprodrug: synthesis, characterization, and supramolecular hydrogel formation for the drug combination. *J. Mater. Chem. B.* 9 3200–3209. 10.1039/d0tb02719g 33885624

[B21] Hamley I. W. (2017). Small bioactive peptides for biomaterials design and therapeutics. *Chem. Rev.* 117 14015–14041. 10.1021/acs.chemrev.7b00522 29227635

[B22] Han H. W. Lin H. Y. He D. L. Ren Y. Sun W. X. Liang L. (2018). Novel podophyllotoxin derivatives as potential tubulin inhibitors: design, synthesis, and antiproliferative activity evaluation. *Chem. Biodivers.* 15:e1800289. 10.1002/cbdv.201800289 30194898

[B23] Han H. W. Qiu H. Y. Hu C. Sun W. X. Yang R. W. Qi J. L. (2016). Design, synthesis and anti-cancer activity evaluation of podophyllotoxin-norcantharidin hybrid drugs. *Bioorg. Med. Chem. Lett.* 26 3237–3242. 10.1016/j.bmcl.2016.05.063 27262599

[B24] Hong W. G. Cho J. H. Hwang S. G. Lee E. Lee J. Kim J. I. (2016). Chemosensitizing effect of podophyllotoxin acetate on topoisomerase inhibitors leads to synergistic enhancement of lung cancer cell apoptosis. *Int. J. Oncol.* 48 2265–2276. 10.3892/ijo.2016.3471 27035096 PMC4863929

[B25] Hu C. Xu D. Du W. Qian S. Wang L. Lou J. (2010). Novel 4 beta-anilino-podophyllotoxin derivatives: design synthesis and biological evaluation as potent DNA-topoisomerase II poisons and anti-MDR agents. *Mol. Biosyst.* 6 410–420. 10.1039/b912336a 20094661

[B26] Hui L. Sang C. Wang D. Wang X. Wang M. Jia Q. (2016). Newly synthesized podophyllotoxin derivative, LJ12, induces apoptosis and mitotic catastrophe in non-small cell lung cancer cells in vitro. *Mol. Med. Rep.* 13 339–346. 10.3892/mmr.2015.4561 26573436

[B27] Hyder I. Yedlapudi D. Kalivendi S. V. Khazir J. Ismail T. Nalla N. (2015). Synthesis and biological evaluation of novel 4β-[(5-substituted)-1,2,3,4-tetrazolyl] podophyllotoxins as anticancer compounds. *Bioorg. Med. Chem. Lett.* 25 2860–2863. 10.1016/j.bmcl.2015.04.053 26022842

[B28] Kamal A. Nayak V. L. Bagul C. Vishnuvardhan M. V. Mallareddy A. (2015). Investigation of the mechanism and apoptotic pathway induced by 4β cinnamido linked podophyllotoxins against human lung cancer cells A549. *Apoptosis* 20 1518–1529. 10.1007/s10495-015-1173-6 26386574

[B29] Kamal A. Suresh P. Janaki Ramaiah M. Mallareddy A. Kumar B. A. Raju P. (2011a). Synthesis and biological evaluation of 4β-acrylamidopodophyllotoxin congeners as DNA damaging agents. *Bioorg. Med. Chem.* 19 4589–4600. 10.1016/j.bmc.2011.06.017 21737288

[B30] Kamal A. Suresh P. Mallareddy A. Kumar B. A. Reddy P. V. Raju P. (2011b). Synthesis of a new 4-aza-2,3-didehydropodophyllotoxin analogues as potent cytotoxic and antimitotic agents. *Bioorg. Med. Chem.* 19 2349–2358. 10.1016/j.bmc.2011.02.020 21402478

[B31] Kamal A. Tamboli J. R. Nayak V. L. Adil S. F. Vishnuvardhan M. V. Ramakrishna S. (2014). Synthesis of a terphenyl substituted 4-aza-2,3-didehydropodophyllotoxin analogues as inhibitors of tubulin polymerization and apoptosis inducers. *Bioorg. Med. Chem.* 22 2714–2723. 10.1016/j.bmc.2014.03.021 24721832

[B32] Kamal A. Tamboli J. R. Ramaiah M. J. Adil S. F. Pushpavalli S. N. Ganesh R. (2013). Quinazolino linked 4β-amidopodophyllotoxin conjugates regulate angiogenic pathway and control breast cancer cell proliferation. *Bioorg. Med. Chem.* 21 6414–6426. 10.1016/j.bmc.2013.08.051 24055291

[B33] Kandil S. Wymant J. M. Kariuki B. M. Jones A. T. McGuigan C. Westwell A. D. (2016). Novel cis-selective and non-epimerisable C3 hydroxy azapodophyllotoxins targeting microtubules in cancer cells. *Eur. J. Med. Chem.* 110 311–325. 10.1016/j.ejmech.2015.12.037 26854430 PMC4762250

[B34] Kartal-Yandim M. Adan-Gokbulut A. Baran Y. (2016). Molecular mechanisms of drug resistance and its reversal in cancer. *Crit. Rev. Biotechnol.* 36 716–726. 10.3109/07388551.2015.1015957 25757878

[B35] Katoch A. Nayak D. Faheem M. M. Kumar A. Sahu P. K. Gupta A. P. (2021). Natural podophyllotoxin analog 4DPG attenuates EMT and colorectal cancer progression via activation of checkpoint kinase 2. *Cell Death Discov.* 7 25. 10.1038/s41420-021-00405-3 33500399 PMC7838189

[B36] Khaled M. Belaaloui G. Jiang Z. Z. Zhu X. Zhang L. Y. (2016). Antitumor effect of Deoxypodophyllotoxin on human breast cancer xenograft transplanted in BALB/c nude mice model. *J. Infect. Chemother.* 22 692–696. 10.1016/j.jiac.2016.07.017 27578026

[B37] Kim B. Srivastava S. K. Kim S. H. (2015). Caspase-9 as a therapeutic target for treating cancer. *Expert Opin. Ther. Targets* 19 113–127. 10.1517/14728222.2014.961425 25256701

[B38] Kim J. Y. Cho J. H. Choi J. R. Shin H. J. Song J. Y. Hwang S. G. (2018). A novel anti-cancer role of β-apopicropodophyllin against non-small cell lung cancer cells. *Toxicol. Appl. Pharmacol.* 357 39–49. 10.1016/j.taap.2018.08.022 30170025

[B39] Kinner A. Wu W. Staudt C. Iliakis G. (2008). Gamma-H2AX in recognition and signaling of DNA double-strand breaks in the context of chromatin. *Nucleic Acids Res.* 36 5678–5694. 10.1093/nar/gkn550 18772227 PMC2553572

[B40] Kwak A. W. Yoon G. Lee M. H. Cho S. S. Shim J. H. Chae J. I. (2020). Picropodophyllotoxin, an epimer of podophyllotoxin, causes apoptosis of human esophageal squamous cell carcinoma cells through ros-mediated JNK/P38 MAPK Pathways. *Int. J. Mol. Sci.* 21 4640. 10.3390/ijms21134640 32629820 PMC7369713

[B41] Li Y. Chen M. Yao B. Lu X. Zhang X. He P. (2019a). Transferrin receptor-targeted redox/pH-sensitive podophyllotoxin prodrug micelles for multidrug-resistant breast cancer therapy. *J. Mater. Chem. B.* 7 5814–5824. 10.1039/c9tb00651f 31495855

[B42] Li Y. Huang T. Fu Y. Wang T. Zhao T. Guo S. (2019b). Antitumor activity of a novel dual functional podophyllotoxin derivative involved PI3K/AKT/mTOR pathway. *PLoS One* 14:e0215886. 10.1371/journal.pone.0215886 31557166 PMC6763125

[B43] Li Y. Wang T. Sun Y. Huang T. Li C. Fu Y. (2019c). p53-mediated PI3K/AKT/mTOR pathway played a role in Ptox(Dpt)-induced EMT inhibition in liver cancer cell lines. *Oxid. Med. Cell Longev.* 2019:2531493. 10.1155/2019/2531493 31191795 PMC6525883

[B44] Lin D. C. Mayakonda A. Dinh H. Q. Huang P. Lin L. Liu X. (2017). Genomic and epigenomic heterogeneity of hepatocellular carcinoma. *Cancer Res.* 77 2255–2265. 10.1158/0008-5472.Can-16-2822 28302680 PMC5413372

[B45] Manzari M. T. Shamay Y. Kiguchi H. Rosen N. Scaltriti M. Heller D. A. (2021). Targeted drug delivery strategies for precision medicines. *Nat. Rev. Mater.* 6 351–370. 10.1038/s41578-020-00269-6 PMC8691416 34950512

[B46] Nayak D. Kumar A. Chakraborty S. Rasool R. U. Amin H. Katoch A. (2017). Inhibition of Twist1-mediated invasion by Chk2 promotes premature senescence in p53-defective cancer cells. *Cell Death Differ.* 24 1275–1287. 10.1038/cdd.2017.70 28498365 PMC5520175

[B47] Oh H. N. Kwak A. W. Lee M. H. Kim E. Yoon G. Cho S. S. (2021). Targeted inhibition of c-MET by podophyllotoxin promotes caspase-dependent apoptosis and suppresses cell growth in gefitinib-resistant non-small cell lung cancer cells. *Phytomedicine* 80:153355. 10.1016/j.phymed.2020.153355 33039730

[B48] Pan C. H. Otsuka Y. Sridharan B. Woo M. Leiton C. V. Babu S. (2020). An unbiased high-throughput drug screen reveals a potential therapeutic vulnerability in the most lethal molecular subtype of pancreatic cancer. *Mol. Oncol.* 14 1800–1816. 10.1002/1878-0261.12743 32533886 PMC7400780

[B49] Passarella D. Giardini A. Peretto B. Fontana G. Sacchetti A. Silvani A. (2008). Inhibitors of tubulin polymerization: synthesis and biological evaluation of hybrids of vindoline, anhydrovinblastine and vinorelbine with thiocolchicine, podophyllotoxin and baccatin III. *Bioorg. Med. Chem.* 16 6269–6285. 10.1016/j.bmc.2008.04.025 18468444

[B50] Pasut G. Veronese F. M. (2009). PEG conjugates in clinical development or use as anticancer agents: an overview. *Adv. Drug Deliv. Rev.* 61 1177–1188. 10.1016/j.addr.2009.02.010 19671438

[B51] Paz-Ares L. Dvorkin M. Chen Y. Reinmuth N. Hotta K. Trukhin D. (2019). Durvalumab plus platinum-etoposide versus platinum-etoposide in first-line treatment of extensive-stage small-cell lung cancer (CASPIAN): a randomised, controlled, open-label, phase 3 trial. *Lancet* 394 1929–1939. 10.1016/s0140-6736(19)32222-6 31590988

[B52] Peña-Morán O. A. Villarreal M. L. Álvarez-Berber L. Meneses-Acosta A. Rodríguez-López V. (2016). Cytotoxicity, post-treatment recovery, and selectivity analysis of naturally occurring podophyllotoxins from bursera fagaroides var. fagaroides on breast cancer cell lines. *Molecules* 21:1013. 10.3390/molecules21081013 27527135 PMC6274026

[B53] Porter A. G. Jänicke R. U. (1999). Emerging roles of caspase-3 in apoptosis. *Cell Death Differ.* 6 99–104. 10.1038/sj.cdd.4400476 10200555

[B54] Reddy V. G. Bonam S. R. Reddy T. S. Akunuri R. Naidu V. G. M. Nayak V. L. (2018). 4β-amidotriazole linked podophyllotoxin congeners: DNA topoisomerase-IIα inhibition and potential anticancer agents for prostate cancer. *Eur. J. Med. Chem.* 144 595–611. 10.1016/j.ejmech.2017.12.050 29289884

[B55] Ren J. Liu Y. Li L. Zhao Y. Li Z. Wu C. (2018). OAMDP, a novel podophyllotoxin derivative, induces apoptosis, cell cycle arrest and autophagy in hepatoma HepG2 cells. *Cell Biol Int.* 42 194–204. 10.1002/cbin.10892 29052919

[B56] Resendez A. Tailor D. Graves E. Malhotra S. V. (2019). Radiosensitization of Head and Neck Squamous Cell Carcinoma (HNSCC) by a Podophyllotoxin. *ACS Med. Chem. Lett.* 10 1314–1321. 10.1021/acsmedchemlett.9b00270 31531203 PMC6746081

[B57] Roy A. Ernsting M. J. Undzys E. Li S. D. (2015). A highly tumor-targeted nanoparticle of podophyllotoxin penetrated tumor core and regressed multidrug resistant tumors. *Biomaterials* 52 335–346. 10.1016/j.biomaterials.2015.02.041 25818440 PMC4379456

[B58] Roy A. Zhao Y. Yang Y. Szeitz A. Klassen T. Li S. D. (2017). Selective targeting and therapy of metastatic and multidrug resistant tumors using a long circulating podophyllotoxin nanoparticle. *Biomaterials* 137 11–22. 10.1016/j.biomaterials.2017.05.019 28528299 PMC5516947

[B59] Sadeghi I. Behmanesh M. Ahmadian Chashmi N. Sharifi M. Soltani B. M. (2015). 6-Methoxy Podophyllotoxin Induces Apoptosis via Inhibition of TUBB3 and TOPIIA Gene Expressions in 5637 and K562 Cancer Cell Lines. *Cell J.* 17 502–509. 10.22074/cellj.2015.10 26464822 PMC4601871

[B60] Sang C. Y. Xu X. H. Qin W. W. Liu J. F. Hui L. Chen S. W. (2013). DPMA, a deoxypodophyllotoxin derivative, induces apoptosis and anti-angiogenesis in non-small cell lung cancer A549 cells. *Bioorg. Med. Chem. Lett.* 23 6650–6655. 10.1016/j.bmcl.2013.10.048 24231363

[B61] Sathish M. Kavitha B. Nayak V. L. Tangella Y. Ajitha A. Nekkanti S. (2018). Synthesis of podophyllotoxin linked β-carboline congeners as potential anticancer agents and DNA topoisomerase II inhibitors. *Eur. J. Med. Chem.* 144 557–571. 10.1016/j.ejmech.2017.12.055 29289881

[B62] Shankaraiah N. Kumar N. P. Amula S. B. Nekkanti S. Jeengar M. K. Naidu V. G. (2015). One-pot synthesis of podophyllotoxin-thiourea congeners by employing NH*2*SO*3*H/NaI: Anticancer activity, DNA topoisomerase-II inhibition, and apoptosis inducing agents. *Bioorg. Med. Chem. Lett.* 25 4239–4244. 10.1016/j.bmcl.2015.07.100 26292628

[B63] Sharma S. Mehak Chhimwal J. Patial V. Sk U. H. (2019). Dendrimer-conjugated podophyllotoxin suppresses DENA-induced HCC progression by modulation of inflammatory and fibrogenic factors. *Toxicol Res.* 8 560–567. 10.1039/c9tx00103d 31367338 PMC6621132

[B64] Stähelin H. F. von Wartburg A. (1991). The chemical and biological route from podophyllotoxin glucoside to etoposide: ninth Cain memorial Award lecture. *Cancer Res.* 51 5–15. 1988106

[B65] Sun W. X. Ji Y. J. Wan Y. Han H. W. Lin H. Y. Lu G. H. (2017). Design and synthesis of piperazine acetate podophyllotoxin ester derivatives targeting tubulin depolymerization as new anticancer agents. *Bioorg. Med. Chem. Lett.* 27 4066–4074. 10.1016/j.bmcl.2017.07.047 28757065

[B66] Tailor D. Going C. C. Resendez A. Kumar V. Nambiar D. K. Li Y. (2021). Novel Aza-podophyllotoxin derivative induces oxidative phosphorylation and cell death via AMPK activation in triple-negative breast cancer. *Br. J. Cancer* 124 604–615. 10.1038/s41416-020-01137-4 33139797 PMC7851402

[B67] Tummers B. Green D. R. (2017). Caspase-8: regulating life and death. *Immunol. Rev.* 277 76–89. 10.1111/imr.12541 28462525 PMC5417704

[B68] Vander Heiden M. G. Cantley L. C. Thompson C. B. (2009). Understanding the Warburg effect: the metabolic requirements of cell proliferation. *Science* 324 1029–1033. 10.1126/science.1160809 19460998 PMC2849637

[B69] Vishnuvardhan M. Saidi Reddy V. Chandrasekhar K. Lakshma Nayak V. Sayeed I. B. Alarifi A. (2017). Click chemistry-assisted synthesis of triazolo linked podophyllotoxin conjugates as tubulin polymerization inhibitors. *Medchemcomm* 8 1817–1823. 10.1039/c7md00273d 30108892 PMC6084182

[B70] Vodenkova S. Buchler T. Cervena K. Veskrnova V. Vodicka P. Vymetalkova V. (2020). 5-fluorouracil and other fluoropyrimidines in colorectal cancer: past, present and future. *Pharmacol. Ther.* 206:107447. 10.1016/j.pharmthera.2019.107447 31756363

[B71] Wang B. Chen L. Zhen H. Zhou L. Shi P. Huang Z. (2013). Proteomic changes induced by podophyllotoxin in human cervical carcinoma HeLa cells. *Am. J. Chin. Med.* 41 163–175. 10.1142/s0192415x13500122 23336514

[B72] Wang J. Long L. Chen Y. Xu Y. Zhang L. (2018). Design, synthesis and antineoplastic activity of novel hybrids of podophyllotoxin and indirubin against human leukaemia cancer cells as multifunctional anti-MDR agents. *Bioorg. Med. Chem. Lett.* 28 1817–1824. 10.1016/j.bmcl.2018.04.019 29678463

[B73] Wang Y. Sun H. Xiao Z. Zhang D. Bao X. Wei N. (2017). XWL-1-48 exerts antitumor activity via targeting topoisomerase II and enhancing degradation of Mdm2 in human hepatocellular carcinoma. *Sci. Rep.* 7:9989. 10.1038/s41598-017-10577-7 28855652 PMC5577045

[B74] Wang Y. Sun H. Xiao Z. Zhang G. Zhang D. Bao X. (2018). DNA damage and apoptosis induced by a potent orally podophyllotoxin derivative in breast cancer. *Cell Commun. Signal.* 16:52. 10.1186/s12964-018-0263-9 30176902 PMC6122736

[B75] Wilson B. E. Jacob S. Yap M. L. Ferlay J. Bray F. Barton M. B. (2019). Estimates of global chemotherapy demands and corresponding physician workforce requirements for 2018 and 2040: a population-based study. *Lancet Oncol.* 20 769–780. 10.1016/s1470-2045(19)30163-9 31078462

[B76] Wu G. R. Xu B. Yang Y. Q. Zhang X. Y. Fang K. Ma T. (2018). Synthesis and biological evaluation of podophyllotoxin derivatives as selective antitumor agents. *Eur. J. Med. Chem.* 155 183–196. 10.1016/j.ejmech.2018.05.052 29886322

[B77] Yang C. Xie Q. Zeng X. Tao N. Xu Y. Chen Y. (2019). Novel hybrids of podophyllotoxin and formononetin inhibit the growth, migration and invasion of lung cancer cells. *Bioorg. Chem.* 85 445–454. 10.1016/j.bioorg.2019.02.019 30776555

[B78] Yang Y. J. Qi S. N. Shi R. Y. Yao J. Wang L. S. Yuan H. Q. (2017). Induction of apoptotic DNA fragmentation mediated by mitochondrial pathway with caspase-3-dependent BID cleavage in human gastric cancer cells by a new nitroxyl spin-labeled derivative of podophyllotoxin. *Biomed. Pharmacother.* 90 131–138. 10.1016/j.biopha.2017.03.048 28347917

[B79] Zhang H. Xu H. Ashby C. R. Jr. Assaraf Y. G. Chen Z. S. Liu H. M. (2021). Chemical molecular-based approach to overcome multidrug resistance in cancer by targeting P-glycoprotein (P-gp). *Med. Res. Rev.* 41 525–555. 10.1002/med.21739 33047304

[B80] Zhang L. Chen F. Zhang Z. Chen Y. Lin Y. Wang J. (2016a). Design, synthesis and evaluation of the multidrug resistance-reversing activity of pyridine acid esters of podophyllotoxin in human leukemia cells. *Bioorg. Med. Chem. Lett.* 26 4466–4471. 10.1016/j.bmcl.2016.07.072 27503681

[B81] Zhang L. Liu L. Zheng C. Wang Y. Nie X. Shi D. (2017a). Synthesis and biological evaluation of novel podophyllotoxin-NSAIDs conjugates as multifunctional anti-MDR agents against resistant human hepatocellular carcinoma Bel-7402/5-FU cells. *Eur. J Med. Chem.* 131 81–91. 10.1016/j.ejmech.2017.03.011 28301815

[B82] Zhang L. Wang J. Liu L. Zheng C. Wang Y. (2017b). Synthesis and Antiproliferative activity of novel all-trans-retinoic acid-podophyllotoxin conjugate towards human gastric cancer cells. *Molecules* 22:628. 10.3390/molecules22040628 28420180 PMC6154554

[B83] Zhang L. Zhang Z. Chen F. Chen Y. Lin Y. Wang J. (2016b). Aromatic heterocyclic esters of podophyllotoxin exert anti-MDR activity in human leukemia K562/ADR cells via ROS/MAPK signaling pathways. *Eur. J. Med. Chem.* 123 226–235. 10.1016/j.ejmech.2016.07.050 27484511

[B84] Zhang W. Liu C. Li J. Liu R. Zhuang J. Feng F. (2020). Target analysis and mechanism of podophyllotoxin in the treatment of triple-negative breast cancer. *Front. Pharmacol.* 11:1211. 10.3389/fphar.2020.01211 32848800 PMC7427588

[B85] Zhang X. Rakesh K. P. Shantharam C. S. Manukumar H. M. Asiri A. M. Marwani H. M. (2018). Podophyllotoxin derivatives as an excellent anticancer aspirant for future chemotherapy: a key current imminent needs. *Bioorg. Med. Chem.* 26 340–355. 10.1016/j.bmc.2017.11.026 29269253

[B86] Zhao W. Bai J. K. Li H. M. Chen T. Tang Y. J. (2015). Tubulin structure-based drug design for the development of novel 4β-sulfur-substituted podophyllum tubulin inhibitors with anti-tumor activity. *Sci. Rep.* 5:10172. 10.1038/srep10172 25959922 PMC4426677

[B87] Zhao W. Cong Y. Li H. M. Li S. Shen Y. Qi Q. (2020). Challenges and potential for improving the druggability of podophyllotoxin-derived drugs in cancer chemotherapy. *Nat. Prod. Rep.* 38 470–488. 10.1039/d0np00041h 32895676

[B88] Zhao W. He L. Xiang T. L. Tang Y. J. (2019). Discover 4β-NH-(6-aminoindole)-4-desoxy-podophyllotoxin with nanomolar-potency antitumor activity by improving the tubulin binding affinity on the basis of a potential binding site nearby colchicine domain. *Eur. J. Med. Chem.* 170 73–86. 10.1016/j.ejmech.2019.03.006 30878833

[B89] Zhao Y. Wu Z. Zhang Y. Zhu L. (2013). HY-1 induces G(2)/M cell cycle arrest in human colon cancer cells through the ATR-Chk1-Cdc25C and Weel pathways. *Cancer Sci.* 104 1062–1066. 10.1111/cas.12182 23600770 PMC7657130

[B90] Zhou H. Lv S. Zhang D. Deng M. Zhang X. Tang Z. (2018). A polypeptide based podophyllotoxin conjugate for the treatment of multi drug resistant breast cancer with enhanced efficiency and minimal toxicity. *Acta Biomater.* 73 388–399. 10.1016/j.actbio.2018.04.016 29694920

[B91] Zhou M. Li J. Li C. Guo L. Wang X. He Q. (2016). Tertiary amine mediated targeted therapy against metastatic lung cancer. *J. Control Release* 241 81–93. 10.1016/j.jconrel.2016.09.013 27639682

[B92] Zi C. T. Gao Y. S. Yang L. Feng S. Y. Huang Y. Sun L. (2019). Design, synthesis, and biological evaluation of novel biotinylated podophyllotoxin derivatives as potential antitumor agents. *Front. Chem.* 7:434. 10.3389/fchem.2019.00434 31281809 PMC6596340

[B93] Zi C. T. Yang L. Xu F. Q. Dong F. W. Yang D. Li Y. (2018). Synthesis and anticancer activity of dimeric podophyllotoxin derivatives. *Drug Des. Devel. Ther.* 12 3393–3406. 10.2147/dddt.S167382 30349193 PMC6186772

